# Utilization of Crab Waste for Cost-Effective Bioproduction of Prodigiosin

**DOI:** 10.3390/md18110523

**Published:** 2020-10-22

**Authors:** Van Bon Nguyen, Dai Nam Nguyen, Anh Dzung Nguyen, Van Anh Ngo, That Quang Ton, Chien Thang Doan, Thi Phuong Pham, Thi Phuong Hanh Tran, San-Lang Wang

**Affiliations:** 1 Institute of Research and Development, Duy Tan University, Danang 550000, Vietnam; nguyenvanbon2@duytan.edu.vn; 2 Institute of Biotechnology and Environment, Tay Nguyen University, Buon Ma Thuot 630000, Vietnam; dainamnguyen.edu@gmail.com (D.N.N.); nadzungtaynguyenuni@yahoo.com.vn (A.D.N.); ngovananh3979@gmail.com (V.A.N.); 3 Faculty of Chemistry, University of Science, Ho Chi Minh City 700000, Vietnam; ttquang@hcmus.edu.vn; 4 Vietnam National University, Ho Chi Minh City 700000, Vietnam; 5 Department of Science and Technology, Tay Nguyen University, Buon Ma Thuot 630000, Vietnam; dcthang@ttn.edu.vn (C.T.D.); ptphuong@ttn.edu.vn (T.P.P.); ttphanh@ttn.edu.vn (T.P.H.T.); 6 Department of Chemistry, Tamkang University, New Taipei City 25137, Taiwan; 7 Life Science Development Center, Tamkang University, New Taipei City 25137, Taiwan

**Keywords:** *Serratia marcescens*, prodigiosin, bioreactor system, crab shells, bioprocessing, antioxidants, anticancers

## Abstract

This study aimed to establish the culture process for the cost-effective production of prodigiosin (PG) from demineralized crab shell powder (de-CSP), a fishery processing byproduct created via fermentation. Among the tested PG-producing strains, *Serratia marcescens* TNU02 was demonstrated to be the most active strain. Various ratios of protein/de-CSP were used as the sources of C/N for PG biosynthesis. The PG yield was significantly enhanced when the casein/de-CSP ratio was controlled in the range of 3/7 to 4/6. TNU02 produced PG with a high yield (5100 mg/L) in a 15 L bioreactor system containing 4.5 L of a newly-designed liquid medium containing 1.6% C/N source (protein/de-CSP ratio of 3/7), 0.02% (NH_4_)_2_SO_4_, 0.1% K_2_HPO_4_, and an initial pH of 6.15, at 27 °C for 8 h in dark conditions. The red pigment was purified from the culture broth and then quantified as being PG by specific Matrix-Assisted Laser Desorption Ionization-Time of Flight Mass Spectrometry (MALDI-TOF MS) and UV spectra analysis. The purified PG demonstrated moderate antioxidant and effective inhibition against four cancerous cell lines. Notably, this study was the first to report on using crab wastes for PG bioproduction with high-level productivity (5100 mg/L) in a large scale (4.5 L per pilot) in a short period of fermentation time (8 h). The salt compositions, including (NH_4_)_2_SO_4_ and K_2_HPO_4_, were also a novel finding for the enhancement of PG yield by *S. marcescens* in this report.

## 1. Introduction

Crab shells, a marine chitin waste that can be abundantly obtained from fishery processing byproducts, have been used for the production of various bioactive products, including chitin [1,2,3], enzymes [4], coagulants [5] antioxidants [6], and anti-cancer components [7]. Crab shells are also used in Portland cement matrices [8]. Recently, this material has been used for the bioproduction of anti-diabetic agents [9]. In this study, we investigated the use of crab shells for the production of the active medical compound prodigiosin.

**Figure 1:**
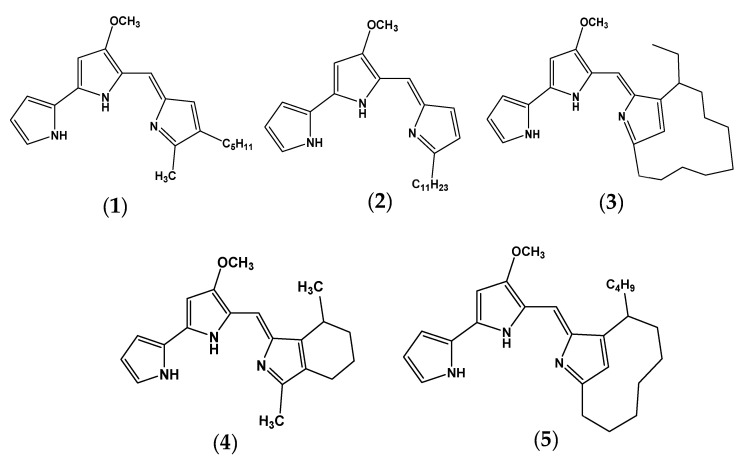
The chemical structures of the pigments belonging to the family of prodiginines. Prodigiosin (**1**), Undecylprodigiosin (**2**), Metacylprodigiosin (**3**), Cycloprodigiosin (**4**), and Streptorubin B (**5**).

Prodigiosin (PG), a microbial red pigment belonging to the family of prodiginines (Figure 1), holds numerous valuable bioactivities and has the potential of being an antiparasitic, antibacterial, anti-inflammatory, andalgicidal, immunosuppressant, antioxidant, and insecticidal agent. PG has also been investigated for its use in textiles, cosmetics, and candles [10], as well as recently for its anti-Alzheimer activities [11]. In addition, PG was newly reclaimed as a novel and useful material for solar cells [12].

This pigment compound is produced by various bacterial strains, including *Serratia rubidaea*, *Alteromonas rubra*, *Janthinobacterium lividum* BR01, *Rugamonas rubra*, *Streptomyces longisporus ruber* 100-19, *S. coelicolor*, *S. spectabilis* BCC 4785, *S. fusant* NRCF69, *V. gazogenes*, *V. psychroerythrus*, *P. magnesiorubra*, *P. putida* KT2440, *S. rubrireticuli*, *P. rubra*, and *Actinomycetes* [10]. Of these, *S. marcescens* is the major producer of PG [11].

Recently, the study of PG has been renewed and increased due to its beneficial effects, especially its high anticancer activity [11,13] and lack of toxicity to normal cells [11]. To enhance the anticancer effect, PG was combined with other agents [14], formed nanoparticle sizes [15], and synthesized its derivatives [16]. Thus, the large-scale production of PG for further clinical studies has been considerable. Numerous studies have focused on PG production [10]; however, most of the C/N sources used for fermentation have been commercial nutrient mediums such as broths of peptone glycerol [17], glycerol/tryptone [18], tryptone yeast, tryptone soy, glycerol, yeast malt [19], nutrient broth (NB) [20], Luria-Bertani (LB) broth, 3-[*N*-morpholino]-ethanesulfonic acid [21], and yeast extract [22]. For the low-cost production of PG via fermentation, several C/N sources have been investigated for fermentation, such as crude glycerol, coconut oil, sesame oil, peanut oil, peanut seed, sesame seed, copra seed, corn steep, and cassava, as well as mixtures of sunflower oil/Luria–Bertani broth, mannitol/corn steep, olive oil/peanut powder/beef extract, and mannitol/cassava [23,24,25,26,27,28].

For the green production of PG, under the considerations of environmental problems and low-cost production, we established the utilization of marine chitin-containing wastes for the production of PG [11,29,30,31]. These previous studies focused on reusing squid pens as a C/N source for the production of PG via fermentation by *S. marcescens* TKU011 [29,30,31] and *S. marcescens* TNU01 [11]. Recently, we found that the addition of chitin to the medium has a significant effect on enhancing PG yield in the culture broth and that α-chitin (extracted from crab shells) has a better effect compared to β-chitin (extracted from squid pens) [32]. This evidence indicated that materials containing α-chitin may be a good source for PG production. Crab shell contains α-chitin as a major component, and as such it was chosen as the target material for the cost-effective production of PG in this investigation.

In this study, crab shell was used as the major C/N source with the supplementation of free protein for fermentation by various strains of *S. marcescens.* This study focused on investigating the most suitable added free protein, the effect of the salt composition, and the optimal conditions for fermentation to produce PG in a 100 mL flash. Production of the PG was then scaled up by using a 15 L bioreactor system for fermentation. Finally, the PG was extracted, isolated from the culture broth and its biological activities were evaluated, including its antioxidant and anticancer effects.

## 2. Results and Discussion

### 2.1. The Effect of Different Added Free Proteins and Differrent Strains of S. marcescens on PG Production

**Figure 2:**
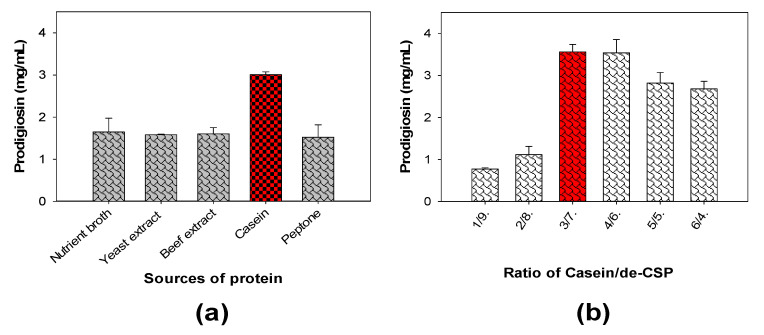
The effect of sources of protein added (**a**) and casein/de-CSP ratio (**b**) on PG production. The C/N was a mixture of 1% crab shell and 0.6% free protein (**a**), or the combination of casein and de-CSP at different ratios and used at 1.6% as the C/N source (**b**) in a basal salt solution of 0.1% CaSO_4_, 0.05% K_2_HPO_4_ and a pH of 6.15 for fermentation by *S. marcescens* TKU011 under the fermentation conditions of 25 °C, a duration of 2 days, a shaking speed of 150 rpm in the dark, and a culture medium/flask volume ratio of 3/7 (*v*/*v*) (30 mL of liquid culture medium in a 100 mL flask). The column with red color indicated that it is the factor chosen for further investigation.

For the investigation of the effect of different protein sources added to a medium containing demineralized crab shell powder (de-CSP) on PG production, five types of protein, including casein, beef extract, nutrient broth, peptone, and yeast extract, were supplemented for fermentation to produce PG. As presented in Figure 2a, casein was the most suitable free protein for PG production, with the highest yield of 3.01 mg/mL. To investigate the optimal added concentration of casein, this protein source was combined with de-CSP at various ratios (Casein/de-CSP) of 1/9, 2/8, 3/7, 4/6, 5/5, and 6/4 then used as the C/N source for fermentation. This sub-experimental result is illustrated in Figure 2b. The casein/de-CSP ratios of 3/7 and 4/6 reached the highest PG yield of ≥3.5 mg/mL. With regard to the utilization of crab waste for the cost-effective production of PG, a casein/de-CSP ratio of 3/7 was conducted for further investigation.

For comparison of the PG production ability of different bacterial strains, four strains of *S. marcescens*, including TKU011, CC17, TNU01, and TNU02 were fermented in the same conditions. The data are summarized as shown in Table 1. The results indicated that three strains, including TKU011, TNU01, and TNU01 were promising PG-producing strains that could produce an equal PG yield of 3.52–3.61 mg/mL. *S. marcescens* TNU02 was chosen for further investigation.

### 2.2. The Effect of Salt Composition and Some Parameters of Fermentation on PG Production

**Figure 3:**
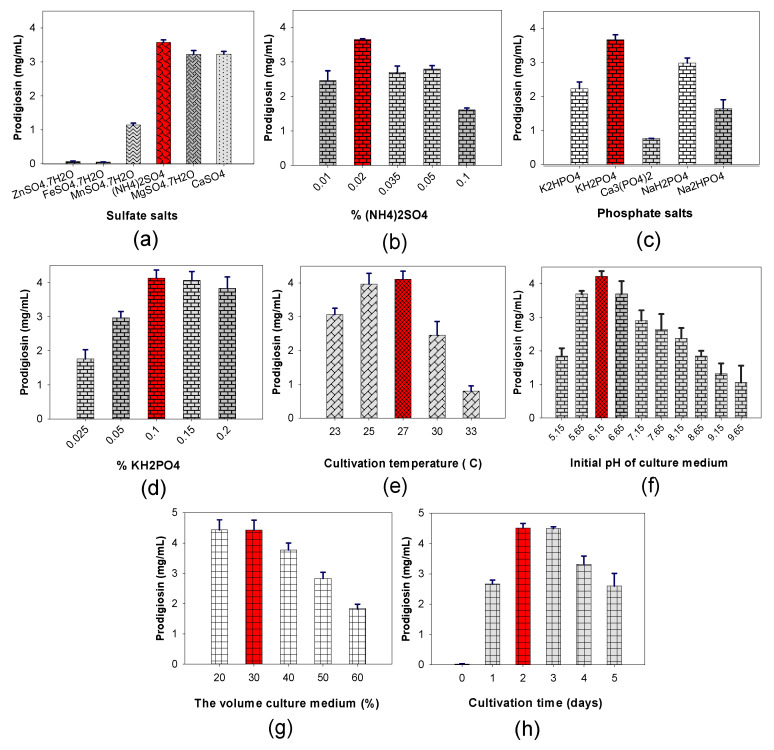
The influence of (**a**) sulfate salts, (**b**) (NH_4_)_2_SO_4_; (**c**) phosphate salts; (**d**) KH_2_PO_4_; (**e**) fermentation temperature; (**f**) initial pH of the culture medium; (**g**) the percentage of culture medium; (**h**) time course of fermentation on PG production by *S. marcescens* TNU02 fermentation. The column with red color indicated that it is the factor chosen for further investigation.

Our previous studies revealed that the salt composition, including sulfate and phosphate salts, demonstrate a significant effect on PG production using *S. marcescens* [11,30,31,32]. Thus, six kinds of sulfate salts (MgSO_4_·7H_2_O, CaSO_4_, ZnSO_4_·7H_2_O, MnSO_4_·7H_2_O, FeSO_4_·7H_2_O, and (NH_4_)_2_SO_4_) and five kinds of phosphate salts (K_2_HPO_4_, KH_2_PO_4_, Ca_3_(PO_4_)_2_, NaH_2_PO_4_, and Na_2_HPO_4_) were conducted to evaluate their effect on PG production via fermentation (Figure 3).

Among the various tested salts, (NH_4_)_2_SO_4_ and KH_2_PO_4_ were screened as the most suitable salt compositions (Figure 3a,c) at the optimal added concentrations of 0.02% and 0.1%, respectively (Figure 3b,d). K_2_HPO_4_ and MgSO_4_ have been found to be the most suitable sources of phosphate and sulfate salts, respectively, in many previous reports [11]. Recently, K_2_HPO_4_ and CaSO_4_ have been found to be the best salt compositions for PG production [32]. However, in this study, KH_2_PO_4_ and (NH_4_)_2_SO_4_ were newly found to play a significant role in PG yield enhancement in the fermentation of *S. marcescens*.

For the maximum PG production by *S. marcescens* TNU02 fermentation, some fermentation parameters, such as temperature, pH of the culture medium, culture volume and cultivation time, were investigated. The results illustrated in Figure 3e–k show that *S. marcescens* TNU02 induced the highest PG yield of 4.51 mg/mL in the designed liquid medium containing a 1.6% C/N source (a casein/de-CSP ratio of 3/7), 0.02% (NH_4_)_2_SO_4_, 0.1% K_2_HPO_4_, an initial pH of 6.15, and a percentage of culture medium in the flask of 30% (30 mL of liquid culture medium in a 100 mL flask), at 27 °C for 2 days in dark conditions. After optimization of the culture conditions, some nutrients and parameters changed and the PG yield dramatically increased by approximately 1.5-fold, from 3.01 mg/mL to 4.51 mg/mL (Table 2).

### 2.3. Scaled up Biosynthesis, Extraction and Qualification of PG Produced by S. marcescens TNU02

**Figure 4:**
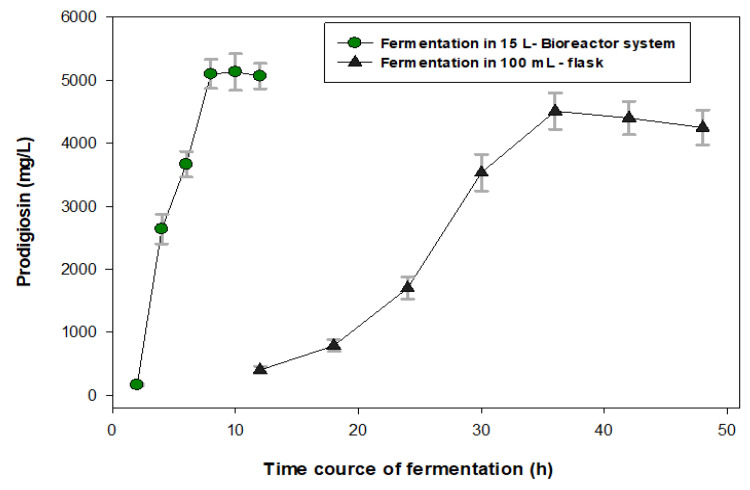
Production of PG by *S. marcescens* TNU02 in a 15 L bioreactor system and in a 100 mL flask. 450 mL of *S. marcescens* TNU02 was previously fermented in a 1000 mL flask for 2 days and then injected into a 15 L bioreactor system containing 4.05 L of a culture medium containing 1.6% C/N source (a casein/de-CSP ratio of 3/7), salt compositions of 0.1% KH_2_PO_4_ and 0.02% (NH_4_)_2_SO_4_, and an initial pH of 6.15. Fermentation was also conducted in a 100 mL flask for comparison. Sampling and determination of the PG concentration was performed every 2 h until 12 h of fermentation had passed in the bioreactor system. Fermentation in the 100 mL flask was also performed at the same time for comparison, and the sampling and determination of the PG concentration was performed every 8 h, up to 48 h during the cultivation.

To achieve the purpose of PG production in mass, we used the optimal culture conditions obtained from the above experiments in a minor scale (a 100 mL flask) to scale up the production of PG by using a 15 L bioreactor system. The production of PG in a 100 mL flask was also performed at the same time for comparison. The PG productivity was detected during fermentation from 2 to 12 h (fermentation in the bioreactor), and for 8 to 48 h (fermentation in the 100 mL flask). As shown in Figure 4, the PG yield reached the maximum (5100 mg/L) at 8 h of fermentation in the 15 L bioreactor system, while the PG yield of fermentation in the 100 mL flask reached the maximum productivity of 4514 mg/L at 36 h of fermentation. Thus, the PG production in the 15 L bioreactor system resulted in reaching a higher PG yield in a much shorter period of fermentation time compared to the PG production in the 100 mL flask. A review of the recent literatures found that numerous studies have reported on PG production. However, in most previous studies, PG production was conducted in a minor scale (in flasks) using commercial nutrients as C/N sources for fermentation [11]. Different to previous research, our study successfully engaged in cost-effective PG production at a large scale (a 15 L bioreactor system) using low-cost material (crab waste) as the C/N source for fermentation. PG was also produced by *S. marcescens* TKU011 in a 10 L bioreactor system with a lower maximal yield (3450 mg/L) at a longer period of fermentation time (12 h) in our previous report [11].

**Figure 5:**
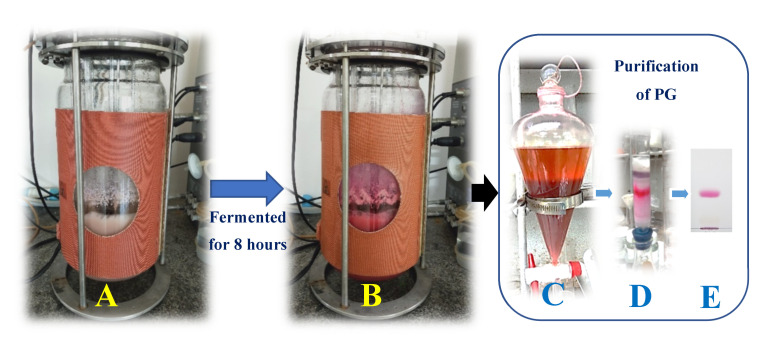
The process of PG production and purification in a 15 L bioreactor system. The liquid medium at the start of fermentation (**A**) turned red after being fermented by *S. marcescens* TNU02 over 8 h (**B**). The red pigment PG was purified via the separation layer by ethyl acetate (**C**) and then further separated in a column containing silica gel (**D**) and was finally isolated by TLC separation (**E**).

**Figure 6:**
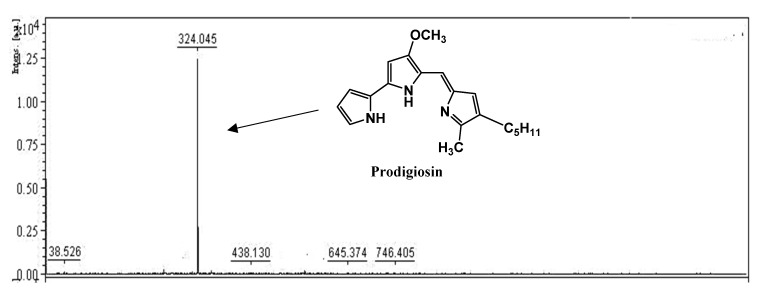
MALDI-TOF MS spectrum of the purified PG produced by *S. marcescens* TNU02 analyzed using the method described in our previous report [32].

**Figure 7:**
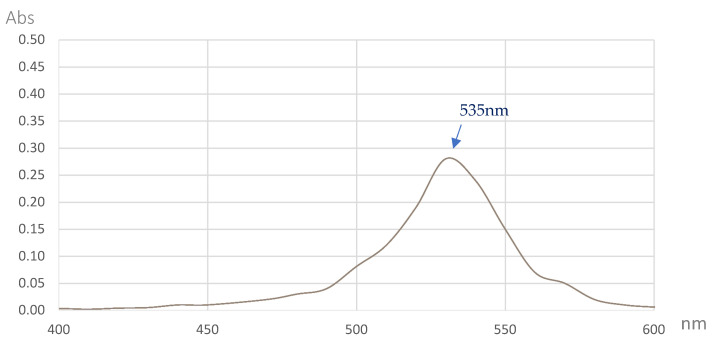
The UV absorption spectrum of *S. marcescens* TNU02 PG produced in a 15 L bioreactor system.

The PG was isolated and purified from the culture broth in a 15 L bioreactor via several steps according to the method previously reported by Wang et al., 2012 [30], including layer separation by using ethyl acetate (Figure 5c) and fractionation by a silica column (Figure 5d), and was finally purified via TLC isolation (Figure 5e). The production, isolation, and purification process of the PG is illustrated in Figure 5. The red purified compound was confirmed as PG using a number of rapid techniques, including its UV absorption and MALDI-TOF MS spectrum. The results shown in Figure 6 and Figure 7 reveal that this red pigment possessed maximal UV absorption at 535 nm and a molecular weight of 323.045 g/mol. These two items represented the specific UV absorption and molecular weight of the PG [30]; thus, this red pigment was confirmed to be PG.

### 2.4. Antioxidant and Anticancers Activities of Purified PG

PG has been investigated for its vast array of medical effects [10]. To confirm that the PG produced by our novel medium with the fermentation of *S. marcescens* TNU02 in a bioreactor system and purified by the reported protocol [30] was active molecular, some biological activities, including the antioxidant and anticancer activities of the PG, were tested.

**Figure 8:**
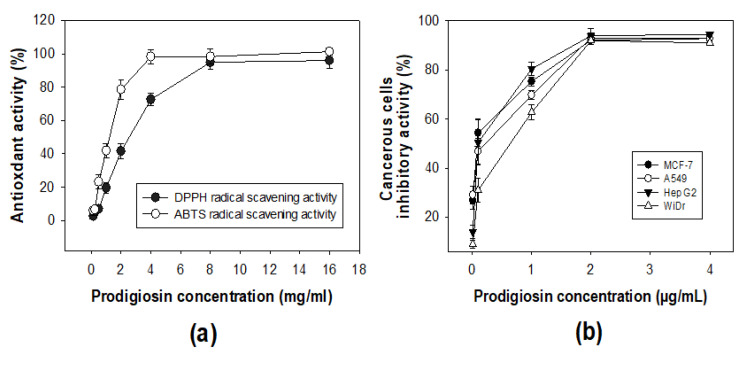
Antioxidant (**a**) and anticancer effects (**b**) of PG produced by *S. marcescens* TNU02 PG in a 15 L bioreactor system.

Antioxidant compounds may protect some major components of cells, such as proteins, DNA, and lipids from the damage of free radicals [33]. In this study, 2,2-diphenyl-1-picrylhydrazyl (DPPH) and 2,2′-Azino-bis(3-ethylbenzothiazoline-6-sulfonic acid) (ABTS) radical scavenging assays were used for evaluation of the antioxidant activity of the PG. As shown in Figure 8a, the PG demonstrated both DPPH and ABTS radical scavenging capacities. Of these, the ABTS radical scavenging activity of the PG was higher than the DPPH radical scavenging capacity of the PG, with a max activity of 98.3% (at a tested PG concentration of 4 mg/mL) and 96% (at a tested PG concentration of 8 mg/mL), respectively. To provide more clarity, the IC_50_ values (the concentration of an antioxidant compound that may reduce 50% of the radical scavenging [11]) of the ABTS and DPPH radical scavenging activity of the PG were also expressed as 1.25 mg/mL and 2.64 mg/mL, respectively. The α-tocopherol, a standard antioxidant compound was tested for the comparison activity and showing its ABTS and DPPH radical scavenging activity of 13.3 µg/mL and 23.1 µg/mL, respectively.

The potential antioxidant capacity of PG has been reported in some studies, such as Muthukumar et al. [34], Arivizhivendhan et al. [35], and Nguyen et al. [11], who found the max activity of the DPPH radical scavenging capacity of PG to be 86%, 99%, and 98%, respectively. However, few data on the ABTS radical scavenging of PG have been reported so far [35]. Notably, almost all of the studies did not report the IC_50_ values on antioxidant activity of PG [11]. Thus, the results of the antioxidant activities reported in this study could support and enrich the available data on the antioxidant effects of PG.

PG has been reported to be effective in inhibiting various cancerous cell lines without being toxic to normal cells; thus, it has been suggested as a potential molecular target in anticancer drugs [36]. To evaluate the inhibitory activity of the PG produced in this study, some cancerous cell lines, including A549, Hep G2, MCF-7, and WiDr, were investigated and tested. As illustrated in Figure 8b, the PG demonstrated a high inhibitory effect with max inhibition values of 92.1%, 93.1%, 94%, and 92% against all the tested cell lines (MCF-7, A549, Hep G2, and WiDr), respectively. To clarify the result, the IC_50_ values were also calculated and showed that the PG possessed potent anticancer activity against MCF-7, A549, Hep G2, and WiDr with the low IC_50_ values of 0.102 µg/mL, 0.182 µg/mL, 0.161 µg/mL, and 0.441 µg/mL, respectively. For comparison, the anticancer activity of the anticancer drug Mitomycin was tested and showed an inhibition effect against MCF-7, A549, Hep G2, and WiDr with max inhibition values of 93.7%, 92.8%, 91.3%, and 93.1% and IC_50_ values of 0.12 µg/mL, 0.11 µg/mL, 0.14 µg/mL, and 0.13 µg/mL, respectively. This comparison indicated that the PG showed comparable anticancer activity against MCF-7, A549, and Hep G2, but weaker inhibition against WiDr than Mitomycin. PG has been reported to have effective inhibition against numerous cancerous cell lines, including MCF-7, A549, and Hep G2 in many studies. However, the effect of PG against WiDr has been rarely reported [32]. The result of the biological activities in this study confirmed that the purified PG produced by *S. marcescens* TNU02 PG was active molecule with potential anticancer properties.

## 3. Materials and Methods

### 3.1. Materials

The PG-producing bacterial strains, including *S. marcescens* TKU011 [31], *S. marcescens* TNU01, *S. marcescens* TNU02 [32], and *S. marcescens* CC17 [37], were provided from our previous studies [31,32,37]. The marine byproduct, crab shells, were required from Shin-Ma Frozen Food Co. (I-Lan, Taiwan) and processed into demineralized crab shell powder (de-CSP) by the method presented in detail by Wang and Yeh [38]. The four cell lines, including MCF-7, A549, Hep G2, and WiDr, were provided by the Bioresources Collection and Research Centre (Hsinchu, Taiwan). All other chemicals and reagents used were of the highest grade available.

### 3.2. Methods

#### 3.2.1. Production of PG via Microbial Fermentation

The effect of different free protein combinations with CSP and different strains of S. marcescens on PG production: Five types of protein, including casein, beef extract, nutrient broth, peptone, and 0.6% yeast extract were supplemented in a culture medium containing 1% de-CSP, 0.1% CaSO_4_, 0.05% K_2_HPO_4_, and a pH of 6.15. This mixture medium was fermented by *Serratia marcescens* TKU011 at 25 °C under a shaking speed of 150 rpm in the dark for 2 days. The PG produced in the culture broth was the determined its con centration. Casein was found as the most suitable protein source to be added. Thus, casein was combined with de-CSP at various ratios of 1/9, 2/8, 3/7, 4/6, 5/5, and 6/4 and used as the C/N source for fermentation at 1.6% for investigation on PG production. The fermentation process was performed under the above conditions using *S. marcescens* TKU011. The optimal casein/de-CSP ratio of 3/7 was used for fermentation by different strains of *S. marcescens*, including *S. marcescens* TKU011, *S. marcescens* TNU01, *S. marcescens* TNU02, and *S. marcescens* CC17 under the same conditions of fermentation for PG production. *S. marcescens* TNU02 was used for the following experiments.

The effect of sulfate salts on PG production: Six kinds of sulfate salts (MgSO_4_·7H_2_O, CaSO_4_, ZnSO_4_·7H_2_O, MnSO_4_·7H_2_O, FeSO_4_·7H_2_O, and (NH_4_)_2_SO_4_) were tested for their effect on PG yield production. The liquid medium contained 0.1% sulfate salt and 0.05% K_2_HPO_4_ and had a pH of 6.15 and a 1.6% C/N source (casein/de-CSP = 3/7). These designed mediums were fermented at 25 °C under a shaking speed of 150 rpm in the dark for 2 days. (NH_4_)_2_SO_4_ expressed the best effect on PG production. This salt was added into the medium at various concentrations of 0.01, 0.02, 0.035, 0.05, and 0.1%. The fermentation was performed with no changes to the other factors so as to investigate the optimal added concentration of (NH_4_)_2_SO_4_.

The effect of phosphate salts on PG production: Five kinds of phosphate salts (K_2_HPO_4_, KH_2_PO_4_, Ca_3_(PO_4_)_2_, NaH_2_PO_4_, Na_2_HPO_4_) were investigated to evaluate their effect on PG production. The liquid medium contained 0.02% (NH_4_)_2_SO_4_ and 0.05% phosphate salt and had a pH 6.15 and a 1.6% C/N source (casein/de-CSP = 3/7). These designed mediums were fermented at 25 °C under a shaking speed of 150 rpm in the dark for 2 days. KH_2_PO_4_ expressed its best effect on PG production. This salt was added into the medium at various concentrations of 0.025, 0.05, 0.1, 0.15, and 0.2%. The fermentation was performed with no changes to the other factors so as to investigate the optimal added concentration of KH_2_PO_4_.

Effect of some fermentation parameters on PG production: A 1.6% C/N source (casein/de-CSP = 3/7) in a liquid medium containing the optimal salt composition of 0.02% (NH_4_)_2_SO_4_ and 0.1% K_2_HPO_4_ was used to investigate the effect of the following parameters: culture temperature (23, 25, 27, 30, and 33 °C); initial pH of the culture medium (5.15, 5.65, 6.15, 6.65, 7.15, 7.65, 8.15, 8.65, 9.15, and 9.65 pH); percentage of culture medium volume compared to that of its flask (20, 30, 40, 50, and 60%); cultivation time (0, 1, 2, 3, 4, and 5 days).

Scaled up production of PG using a BioFlo/CelliGen 115 15 L bioreactor system (Eppendorf North America, Connecticut, US): The optimal culture conditions obtained from the above experiments were used for the investigation of PG production in mass using a 15 L bioreactor system, in which 450 mL of *S. marcescens* TNU02 was first fermented in a 1000 mL flask for 2 days and then injected into 4.05 L of a liquid culture medium (in the 15 L bioreactor system) containing a 1.6% C/N source (casein/de-CSP ratio of 3/7), 0.1% KH_2_PO_4_ and 0.02% (NH_4_)_2_SO_4_, with an initial pH of 6.15. The sampling and determination of the PG concentration was performed every 2 h until 12 h of fermentation had passed.

#### 3.2.2. Extraction, Purification and Qualification of PG

Qualification of the PG was performed as per the assay earlier presented by Wang et al., 2012 [30]. A mixture containing 4 mL methanol, 0.5 mL fermented medium broth, and 0.5 mL 2% AlK(SO_4_)_2_·12H_2_O was centrifuged at 1400× *g* for 5 min. The supernatant was collected and mixed with 0.5 N HCl in methanol at a ratio of 1/9 and then used for measuring the optical density at 535 nm. The purified PG provided from our previous study [11] was used to establish the standard for converting the optical density at 535 nm into the content of the PG.

The PG was purified from the fermented culture broth (FCB) using the protocol recently described in a previous study [32]. The supernatant harvested from the FCB by centrifugation at 10,000× *g* for 15 min was mixed with ethyl acetate (EA) at a ratio of 1/1. This mixture was kept in a funnel for around 3 h, with shaking occurring every half hour. The EA layer containing red pigment PG was collected and dried to a powder by evaporation of the EA at 55 °C in an oven air drier. The crude PG powder was then separated via a silica open column Geduran^®^ Si 60, size: 0.040–0.063 mm; Merck KGaA, Darmstadt, Germany) and the PG was eluted by using the gradient of solvents system of methanol in chloroform changed from the ratio of 0/10 to 2/8 (*v*/*v*). Finally, the PG was purified via the separation of PG on a TLC (thin layer chromatography) plate. After separation of PG on the TLC plate, the lane with red pigments on the TLC plate was cut into various small pieces, and the PG was then dissolved using methanol. The PG was dried to a powder by evaporation of the methanol in an oven air drier at 55 °C. This isolated PG was then used for the detection of MALDI-TOF MS, UV, and medical effects.

#### 3.2.3. Biological Activity Assays

The purified PG obtained in this study was tested for its antioxidant and anticancer activities. The antioxidant activity was evaluated via DPPH and ABTS radical scavenging capacity assays. Of these, the DPPH radical scavenging capacity was performed following the method recently and described by Nguyen et al., 2020 [39], and the ABTS radical scavenging capacity assay was performed according to the assay presented by Arivizhivendhan et al., 2018 [35]. The anticancer activity was investigated following the protocol mentioned in detail in a previous report [40]. α-Tocopherol and Mitomycin were used as standard compounds for testing antioxidant and anticancer activities, respectively.

## 4. Conclusions

Demineralized crab shell powder (de-CSP) was reused for the cost-effective production of bioactive PG. In this study, PG was notably produced in a large scale (4.5 L per pilot bioreactor) with a high-level PG yield (5100 mg/L) in a short period of fermentation time (8 h). The PG was purified and then quantified by specific MALDI-TOF MS and UV spectra analysis. The PG produced by *S. marcescens* TNU02 and isolated in this study demonstrated moderate antioxidant and effective anticancer activities. The results suggested that de-CSP could be a valuable material for mass and cost-effective PG production.

## Figures and Tables

**Table 1:** PG production by various strains of *Serratia marcescens.*

No.	Bacterial Strains	Prodigiosin (mg/mL)
1	*S. marcescens* TKU011	3.52 ± 0.134
2	*S. marcescens* TNU01	3.59 ± 0.153
3	*S. marcescens* TNU02	3.61 ± 0.163
4	*S. marcescens* CC17	2.73 ± 0.102
	No bacterial strain	-

The liquid medium contained 0.1% CaSO_4_, 0.05% K_2_HPO_4_, 1.6% C/N source (casein/de-CSP = 3/7) and had a pH 6.15. These designed mediums were fermented by four strains of *S. marcescens* at 25 °C at a shaking speed of 150 rpm in the dark for 2 days. (-) No PG production.

**Table 2:** Cultivation conditions for PG production before and after optimization.

Factors	Before Optimization	After Optimization
C/N source	Casein/de-CSP = 6/10	Casein/de-CSP = 3/7
Salts compositions	0.05% K_2_HPO_4_ and 0.1% CaSO_4_	0.1% KH_2_PO_4_ and 0.02% (NH_4_)_2_SO_4_
Cultivation temperature (°C)	25	27
Medium/flask volume ratio	4/10	3/10
Initial pH of medium	6.15	6.15
Fermentation time (days)	3	2
PG Productivity (mg/mL)	3.01	4.51
